# Comparison of Tuberculin Skin Test and Interferon-Gamma Release Assay for Latent Tuberculosis Screening in Healthcare Workers

**DOI:** 10.7759/cureus.117357

**Published:** 2026-10-02

**Authors:** Khalid Alrajhi, Ahmad Algarni, Walaa Abulola, Abdulaziz Alhadad, Saja Fallatah, Mariam Alrajhi, Arwa Ghasib, Hanan Fan, Yusuf Vali

**Affiliations:** 1 Internal Medicine, King Fahad Armed Forces Hospital, Jeddah, SAU; 2 Medicine, Ibn Sina National College for Medical Studies, Jeddah, SAU; 3 Pulmonology, King Fahad Armed Forces Hospital, Jeddah, SAU

**Keywords:** healthcare-worker, interferon gamma release assay, latent tuberculosis infection, occupational health screening, quantiferon-tb gold plus, tuberculin skin test

## Abstract

Background

Latent tuberculosis infection (LTBI) screening in healthcare settings remains crucial for tuberculosis control. Current screening methods include the Mantoux tuberculin skin test (TST) and interferon-gamma release assay (IGRA), but their comparative effectiveness in healthcare worker screening remains unclear.

Objective

This study aims to compare the diagnostic performance of the TST and the IGRA QuantiFERON-TB Gold Plus (QFT-Plus) in the detection of LTBI among healthcare workers at King Fahad Armed Forces Hospital in Jeddah. As no definitive gold standard for LTBI exists, QFT-Plus was used as the reference comparator: the primary objective was to quantify agreement between the two tests, while identification of an ROC (receiver operating characteristic)-derived optimal TST induration threshold was a secondary, exploratory objective.

Methods

A retrospective cohort study was conducted between January and November 2020, analyzing data from immunocompetent employees with documented Bacillus Calmette−Guérin (BCG) vaccination status. TST was performed using the Mantoux technique, and QFT-Plus testing was conducted according to manufacturer protocols. Statistical analysis included determination of sensitivity, specificity, and predictive values and ROC curve analysis. Because no gold standard for LTBI exists, these measures describe agreement with QFT-Plus rather than true diagnostic accuracy. Follow-up data were obtained from electronic medical records to assess progression to active tuberculosis over a five-year period.

Results

Among 201 participants, QFT-Plus and TST identified 11.4% and 38.3% positive cases, respectively. TST demonstrated 87.0% sensitivity and 68.0% specificity compared to QFT-Plus, with positive and negative predictive values of 26.0% and 97.6%, respectively. Receiver operating characteristic (ROC) analysis identified an optimal TST threshold of 10.5 mm, with an AUC (area under the curve) of 0.871 (95% CI: 0.805−0.937). This threshold was derived and assessed in the same dataset without independent validation and is therefore exploratory. Demographic analysis revealed no significant differences in TB positivity rates across age groups, gender, or nationality (p > 0.05). Most participants (99.0%) had documented BCG vaccination, which may explain the higher rate of TST positivity relative to QFT-Plus, consistent with reduced TST specificity in this cohort. Additionally, during a five-year follow-up period, none of the individuals with discordant results (TST-positive/IGRA-negative) progressed to active tuberculosis.

Conclusion

Relative to QFT-Plus, TST showed higher comparator-based sensitivity but lower comparator-based specificity, suggesting potential for over-diagnosis in BCG-vaccinated healthcare workers. Because QFT-Plus is an imperfect comparator rather than a gold standard, and because the 10.5 mm threshold was not independently validated, these findings are preliminary and apply to the healthcare worker cohort studied; they should not be read as definitive diagnostic accuracy or as a generalizable screening recommendation. A five-year follow-up demonstrated no progression to active tuberculosis among individuals with discordant TST-positive/IGRA-negative results.

## Introduction

According to the World Health Organization (WHO), approximately one-quarter of the world’s population is infected with *Mycobacterium tuberculosis*, representing latent tuberculosis infection (LTBI) rather than active disease [1].

The burden of TB extends beyond its immediate health impact, significantly affecting patients’ quality of life through symptomatic presentations, prolonged treatment requirements, and complications associated with both the disease itself and non-adherence to treatment regimens [2,3]. Recognizing this global concern, the United Nations has incorporated TB elimination into its third sustainable development goal (SDG). The target aims to end the global TB epidemic by 2035, with specific milestones of reducing TB incidence by 80% by 2030 and achieving a 90% reduction by 2035. Saudi Arabia has actively participated in this initiative since 2016, implementing the End-TB strategy along with various preventive and therapeutic interventions, including the directly observed treatment, short-course (DOTS) program, to achieve “zero burden related to TB” [4-6].

The cost-effectiveness of TB screening remains a subject of debate within the medical community. A comprehensive systematic review published in 2021, analyzing 27 articles, demonstrated that TB screening programs are most effective when targeting high-prevalence populations, such as HIV cases, while screening other risk groups showed limited evidence of cost-effectiveness [7].

Currently, two primary screening methods are available: the Mantoux tuberculin skin test (TST) and the interferon-gamma release assay (IGRA). A study from Portugal compared TST/IGRA with IGRA screening alone and found the latter to be much more effective, with a minor increase in incremental cost [8]. Another meta-analysis of 17 studies compared TST with IGRA in LTBI diagnosis and found comparable effectiveness for the two methods, except in pediatric populations where IGRA demonstrated superior results [9].

Given the limited research evaluating TST and IGRA for TB screening in Saudi Arabia, particularly in healthcare settings, this study aims to address this knowledge gap. As no definitive gold standard exists for the diagnosis of LTBI, our primary objective was to evaluate the level of agreement between TST and IGRA (QuantiFERON-TB Gold Plus, QFT-Plus) in the detection of LTBI among healthcare workers at King Fahad Armed Forces Hospital (KFAFH) in Jeddah, using QFT-Plus as the reference comparator. Specifically, we aimed to (1) determine the prevalence of LTBI detected by each test, (2) assess the concordance between TST and QFT-Plus results, and (3) describe TST performance characteristics (sensitivity, specificity, and predictive values) relative to QFT-Plus positivity measures that reflect agreement with QFT-Plus rather than diagnostic accuracy against a true reference standard, in order to evaluate the utility of TST as an initial, low-cost screening strategy relative to QFT-Plus. As a secondary, exploratory objective that was not prespecified, we additionally used ROC analysis to identify the TST induration threshold that best discriminated QFT-Plus-positive from QFT-Plus-negative participants within this cohort. As an additional objective, we assessed clinical outcomes of discordant TST-positive/QFT-Plus-negative participants over five years of follow-up, specifically whether they progressed to active tuberculosis.

According to the WHO classification, Saudi Arabia is considered a low-tuberculosis-incidence country [10]. Therefore, most LTBI detected among healthcare workers is likely acquired in their country of origin rather than through occupational exposure within the hospital.

## Materials and methods

Study design

A retrospective cohort study was conducted at King Fahad Armed Forces Hospital (KFAFH) in Jeddah, Saudi Arabia, between January and November 2020. The study compared two screening methods for LTBI: the Mantoux TST and the IGRA using the QFT-Plus.

Study population

The target population comprised employees of KFAFH who underwent annual tuberculosis screening between January and November 2020. Inclusion criteria were immunocompetent status, documented Bacillus Calmette−Guérin (BCG) tuberculosis vaccination status, and no evidence of active TB. Exclusion criteria included unknown BCG vaccination status, immunocompromised status, indeterminate QFT-Plus results, or prior treatment with LTBI.

BCG vaccination status was documented from each participant's occupational health immunization record maintained in the hospital's employee health file and corroborated with the electronic medical record where available; participants without a documented BCG history were classified as unknown BCG status and excluded, rather than relying on scar assessment alone. Immunocompetent status was defined as the absence of any diagnosed immunosuppressive condition or immunosuppressive medication use recorded in the employee health file. Evidence of active TB was excluded based on the absence of compatible symptoms, chest radiographic findings, and microbiological confirmation on routine occupational screening. Indeterminate QFT-Plus results, defined per the manufacturer's criteria (an elevated Nil-tube response or an insufficient Mitogen-minus-Nil response), were excluded from the analytic sample, as were participants with a prior diagnosis or treatment for LTBI, to avoid misclassification of test performance. Potential occupational tuberculosis exposure was assessed through the hospital's infection prevention and control (IPC) department, which maintains a contact-tracing log documenting any reported unprotected exposure of staff to a patient with confirmed or suspected infectious pulmonary tuberculosis; employees identified through this log as having a discrete exposure incident during the study period are referred for targeted post-exposure testing outside the routine annual screening program and were not part of the present analytic sample. Beyond this incident-based contact-tracing mechanism, participants were not further stratified by workplace risk category (e.g., clinical department or degree of patient contact), consistent with WHO's classification of Saudi Arabia as a low-tuberculosis-incidence country, in which most LTBI detected in this population is attributable to acquisition in the country of origin rather than occupational transmission.

Sample size and sampling technique

The sample size was determined based on feasibility and availability of participants, with a minimum target of 200 subjects considered sufficient to evaluate diagnostic performance. A total of 201 healthcare workers met the eligibility criteria and were included in the final analysis. A consecutive sampling technique was applied, in which all eligible employees who underwent both TST and QFT-Plus during the study period were enrolled. A formal a priori power or precision calculation was not performed; the minimum target of 200 participants was set pragmatically based on feasibility and the anticipated number of employees who would complete both TST and QFT-Plus during the study period. Post hoc, the achieved sample of 201 participants yielded reasonably precise estimates for the primary diagnostic accuracy measures (e.g., sensitivity 87.0%, 95% CI: 67.9-95.5%; specificity 68.0%, 95% CI: 60.8-74.4%).

Data collection

The Mantoux TST was administered by trained occupational health nursing staff, who injected 0.1 mL (5 tuberculin units) of purified protein derivative intradermally into the volar aspect of the forearm, producing a discrete wheal at the injection site, in accordance with standard Mantoux technique. Results were interpreted after 48-72 hours, and a TST induration ≥10 mm was considered positive for LTBI in healthcare workers. For QFT-Plus testing, whole blood was collected into the four QFT-Plus tubes (Nil, TB1, TB2, and Mitogen) and processed according to the manufacturer's instructions (Qiagen, Hilden, Germany), with interferon-gamma concentration measured by enzyme-linked immunosorbent assay (ELISA); results were classified as positive, negative, or indeterminate using the manufacturer-defined cutoff of an interferon-gamma response ≥0.35 IU/mL and ≥25% above the Nil control, and indeterminate results were excluded as noted above. For each participant, QFT-Plus blood was drawn at the same clinic visit as TST placement, and in all cases before the TST induration was read; this sequence follows standard practice for paired TST/IGRA comparison studies and avoids the possibility that a positive TST reaction could non-specifically boost a subsequent IGRA response. The two tests were interpreted independently of one another: TST induration was measured by trained occupational health nursing staff at 48-72 hours without reference to the QFT-Plus result, and QFT-Plus results were determined in the laboratory using the standardized manufacturer-defined cutoff without knowledge of the corresponding TST outcome.

Statistical analysis

Data analysis was performed using IBM SPSS Statistics for Windows, Version 23 (Released 2015; IBM Corp., Armonk, New York). The Shapiro−Wilk test was used to evaluate the normal distribution of continuous data. Continuous variables are presented as mean ± SD (minimum−maximum), while categorical data are expressed as frequencies and percentages. Between-group comparisons of continuous variables were conducted using unpaired t-tests, and categorical data were compared using Pearson’s chi-square test. Sensitivity, specificity, positive predictive value (PPV), and negative predictive value (NPV) were calculated for the Mantoux (TST) test. Receiver operating characteristic (ROC) analysis was performed to determine optimal thresholds for TB diagnosis using Mantoux measurements, with area under the curve (AUC) and 95% confidence intervals reported. The optimal TST induration cutoff was identified as the point on the ROC curve maximizing the Youden index (sensitivity + specificity − 1). Ninety-five percent confidence intervals for sensitivity, specificity, PPV, and NPV were calculated using the Wilson score method for binomial proportions. Statistical significance was set at p < 0.05.

For comparative analysis, QFT-Plus was prespecified as the comparator. Because no definitive reference standard exists for LTBI, the sensitivity, specificity, predictive values, and ROC measures reported for TST describe agreement with QFT-Plus in this cohort and should not be interpreted as true diagnostic accuracy against confirmed LTBI.

Follow-up assessment

For participants with discordant (TST-positive/QFT-Plus-negative) results, progression to active tuberculosis was ascertained by review of the hospital's electronic medical record for the five years following testing, with incident active TB defined by the same combination of compatible symptoms, chest radiographic findings, and microbiological confirmation used to exclude active disease at baseline. This retrospective, record-based approach captures encounters within the KFAFH system; it could not verify whether a participant received a diagnosis of active tuberculosis, or preventive therapy for LTBI, at another facility outside this system, and data on preventive LTBI therapy uptake among discordant participants were not systematically extracted for this analysis. These points are noted as limitations below.

Ethical considerations

The study was conducted after obtaining institutional review board (IRB) approval from the KFAFH Research Ethics Committee (REC No. 418). All data were collected from electronic medical records and anonymized during collection and analysis to ensure participant confidentiality. No personal identifiers were included in the research database, and access to the data was restricted to the research team members only. As this was a retrospective observational study with no direct patient contact or intervention, it posed minimal risk to the study population. This study adhered to the principles of the Declaration of Helsinki and followed Good Clinical Practice guidelines for medical research.

## Results

During a five-year follow-up period, none of the participants with a negative QFT-Plus test developed active tuberculosis disease. Additionally, none of the individuals with discordant results (TST-positive/IGRA-negative) progressed to active tuberculosis during the same follow-up period.

A total of 201 healthcare workers were included in the study. Table 1 shows the demographic characteristics of the study participants. The mean age was 37.43 years (range: 25−69 years), with no significant difference between TB-negative and TB-positive cases (p = 0.625). Most participants were aged 20−35 years (51.2%), followed by 35−50 years (39.8%) and over 50 years (9.0%), with no significant difference between TB status groups (p = 0.721). Most participants were from the Western Pacific Region (WPR) (71.6%), followed by the Eastern Mediterranean Region (EMR)/non-Saudi (10.9%), EMR/Saudi (8.5%), South-East Asian Region (SEAR) (8.0%), and European Region (EUR) (1.0%), with no significant difference between groups (p = 0.741). By nationality, the majority were Filipino (71.1%), followed by Saudi (8.5%), Indian (7.5%), Egyptian (7.5%), Pakistani (2.0%), and other nationalities (0.5% each, including Irish, Jordanian, Malaysian, Palestinian, German, Sudanese, and Bangladeshi), with no significant difference between groups (p = 0.941).

**Table 1:** Demographic characteristics and distribution of study participants stratified by QFT-Plus results (N = 201). Data are presented as mean ± SD (range) for continuous variables and n (%) for categorical variables. Statistical significance was set at p < 0.05. WHO, World Health Organization. WPR, Western Pacific Region. EMR, Eastern Mediterranean Region. SEAR, South-East Asian Region. EUR, European Region.

Characteristics	All participants (N = 201)	Negative (n = 178)	Positive (n = 23)	P-value
Age (years)	37.4 ± 8.7 (25−69)	37.3 ± 8.6 (25−69)	38.3 ± 9.6 (26−59)	0.625
Age category				
20−35 years	103 (51.2%)	91 (51.1%)	12 (52.2%)	0.721
> 35−50 years	80 (39.8%)	72 (40.4%)	8 (34.8%)	
> 50 years	18 (9.0%)	15 (8.4%)	3 (13%)	
Gender				
Female	140 (69.7%)	124 (69.7%)	16 (69.6%)	0.582
Male	61 (30.3%)	54 (30.3%)	7 (30.4%)	
WHO regional countries				
WPR	144 (71.6%)	127 (71.3%)	17 (73.9%)	0.741
EMR/non-Saudi	22 (10.9%)	21 (11.8%)	1 (4.3%)	
EMR/Saudi	17 (8.5%)	14 (7.9%)	3 (13.0%)	
SEAR	16 (8%)	14 (7.9%)	2 (8.7%)	
EUR	2 (1%)	2 (1.1%)	-	
Nationality				
Filipino	143 (71.1%)	126 (70.8%)	17 (73.9%)	0.941
Saudi	17 (8.5%)	14 (7.9%)	3 (13.0%)	
Indian	15 (7.5%)	13 (7.3%)	2 (8.7%)	
Egyptian	15 (7.5%)	15 (8.4%)	-	
Pakistani	4 (2%)	3 (1.7%)	1 (4.3%)	
Irish	1 (0.5%)	1 (0.6%)	-	
Jordanian	1 (0.5%)	1 (0.6%)	-	
Malaysian	1 (0.5%)	1 (0.6%)	-	
Palestinian	1 (0.5%)	1 (0.6%)	-	
German	1 (0.5%)	1 (0.6%)	-	
Sudanese	1 (0.5%)	1 (0.6%)	-	
Bangladesh	1 (0.5%)	1 (0.6%)	-	

The predominance of healthcare workers originating from high-TB-burden countries, particularly the Philippines, likely explains the higher prevalence of LTBI in this cohort, supporting the theory that LTBI in healthcare workers in Saudi Arabia is primarily imported rather than occupationally acquired.

The QFT-Plus results showed 178 negative cases (88.6%) and 23 positive cases (11.4%). The TST results revealed 124 negative cases (61.7%) and 77 positive cases (38.3%). BCG vaccination status showed that 199 cases (99.0%) were vaccinated (Table 2).

**Table 2:** Distribution of QFT-Plus and TST outcomes and BCG vaccination status among healthcare workers (N = 201). QFT-Plus, QuantiFERON-TB Gold Plus, interferon-gamma release assay (IGRA). TST, Tuberculin Skin Test, Mantoux test. BCG, Bacillus Calmette−Guérin (tuberculosis vaccine).

Test	Value
QFT-Plus	
Negative	178 (88.6%)
Positive	23 (11.4%)
TST (mm)	
Negative (< 10)	124 (61.7%)
Positive (≥ 10)	77 (38.3%)
BCG vaccinated	
Yes	199 (99.0%)
No	2 (1.0%)

In a comparison of TST and QFT-Plus test results (Table 3), among the 23 QFT-Plus-positive cases, 20 were also TST-positive, and three were TST-negative (sensitivity: 87.0%). Of the 178 QFT-Plus-negative cases, 57 were TST-positive, and 121 were TST-negative (specificity: 68.0%).

**Table 3:** Comparison of QFT-Plus and TST results among healthcare workers (N = 201). Values are presented as n (%). QFT-Plus, QuantiFERON-TB Gold Plus, interferon-gamma release assay (IGRA). TST, Mantoux Tuberculin Skin Test.

TST	QFT-Plus		Total
	Positive (n = 23)	Negative (n = 178)	
Positive (n = 77)	20 (87.0%)	57 (32.0%)	77
Negative (n = 124)	3 (13.0%)	121 (68.0%)	124

Analysis of the diagnostic performance metrics revealed that TST results were concordant with QFT-Plus in 20 positive and 121 negative participants, while 57 participants were TST-positive/QFT-Plus-negative and three were TST-negative/QFT-Plus-positive; because neither test is a gold standard, these discordant results are not necessarily false positives or false negatives. Based on these results, the test demonstrated a high sensitivity of 87.0% (95% CI: 67.9-95.5%) and moderate specificity of 68.0% (95% CI: 60.8-74.4%). The PPV was relatively low at 26.0% (95% CI: 17.5-36.7%), while the NPV was excellent at 97.6% (95% CI: 93.1-99.2%) (Table 4).

**Table 4:** Diagnostic performance metrics of TST compared to QFT-Plus (N = 201). QFT-Plus, QuantiFERON-TB Gold Plus. TST, Mantoux Tuberculin Skin Test.

Assessment	Value
Sensitivity	87.0% (95% CI: 67.9–95.5%)
Specificity	68.0% (95% CI: 60.8–74.4%)
Positive predictive value (PPV)	26.0% (95% CI: 17.5–36.7%)
Negative predictive value (NPV)	97.60% (95% CI: 93.1–99.2%)

As a direct measure of agreement between the two tests, Cohen's kappa calculated from the 2×2 comparison above was 0.27 (95% CI: 0.12-0.43), indicating only fair agreement beyond chance (Landis and Koch benchmarks) and reinforcing that TST and QFT-Plus are not interchangeable measures of the same underlying state.

At the optimal threshold, TST showed 82.6% agreement with QFT-Plus-positive results (95% CI: 62.9-93.0%) and 70.2% agreement with QFT-Plus-negative results (95% CI: 63.1-76.5%). The AUC therefore reflects discrimination relative to the QFT-Plus comparator, not definitive discrimination between LTBI and no LTBI (Figure 1).

**Figure 1:**
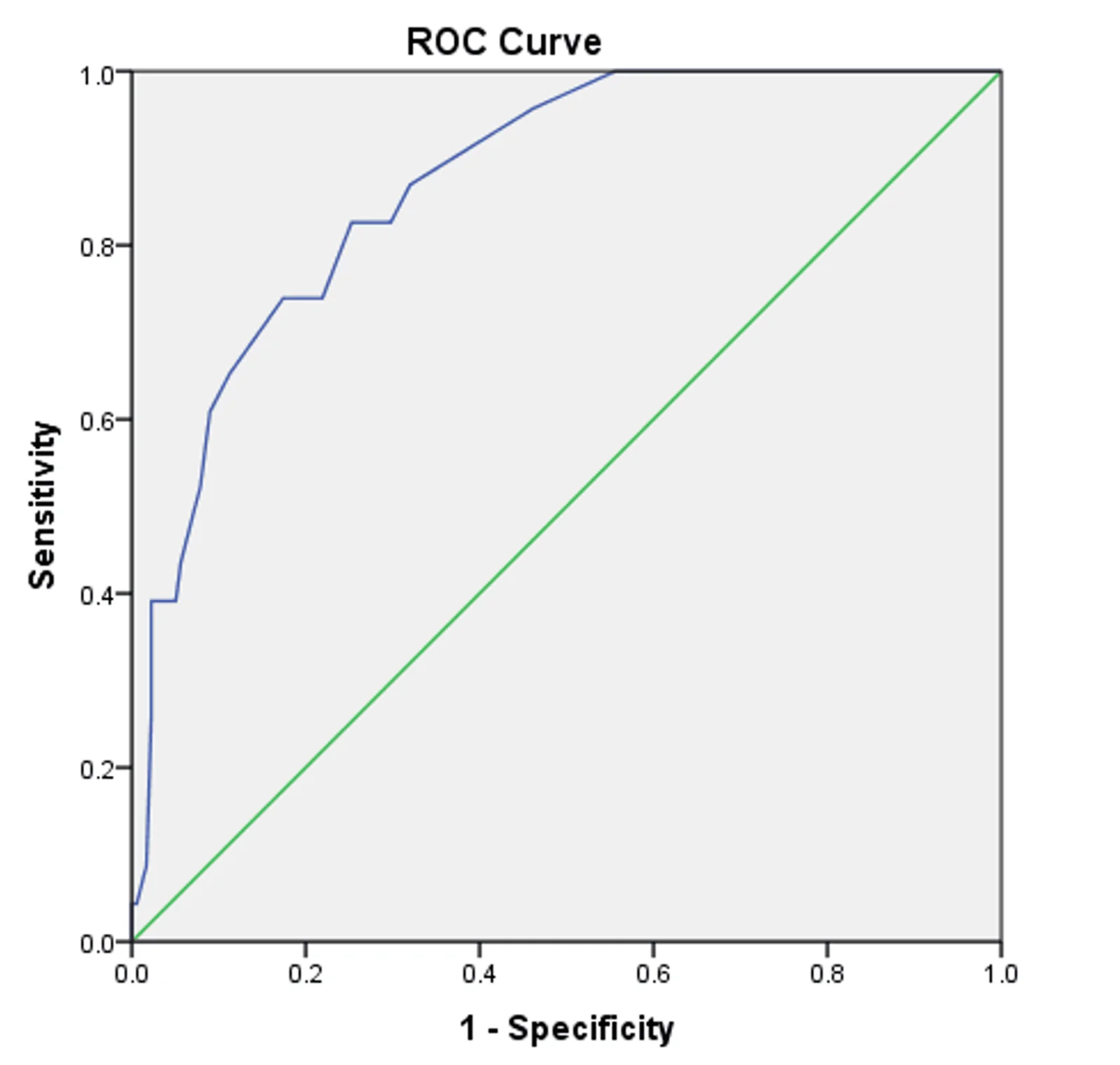
Receiver operating characteristic (ROC) curve analysis comparing QuantiFERON-TB Gold Plus (QFT-Plus, IGRA) and Mantoux tuberculin skin test (TST) performance for detection of latent tuberculosis infection among healthcare workers (N = 201). Area under the curve (AUC): 0.871 (95% CI: 0.805−0.937). Optimal threshold: 10.5 mm induration. Sensitivity: 82.6% (95% CI: 62.9–93.0%); specificity: 70.2% (95% CI: 63.1–76.5%). Figure generated using IBM SPSS Statistics for Windows, Version 23 (Released 2015; IBM Corp., Armonk, New York).

The standard 10 mm induration cutoff (sensitivity 87.0%, specificity 68.0%; Table 4) represents the primary diagnostic performance estimate reported in this study, as this is the internationally recommended threshold for TST interpretation; the ROC-derived 10.5 mm threshold (sensitivity: 82.6%, specificity: 70.2%) is reported as a secondary, exploratory analysis identifying the statistically optimal cutoff within this cohort.

## Discussion

Importantly, none of the individuals with TST-positive/IGRA-negative results developed active tuberculosis over a five-year follow-up period. However, this finding should be interpreted cautiously because the absence of progression to active tuberculosis does not confirm the absence of LTBI, and the number of discordant cases was limited.

The study comprised 201 healthcare workers assessed for LTBI using the Mantoux TST test and the IGRA (QFT-Plus) test. The average age was 37.43 years, and there was no significant difference in age, gender, nationality, or regional distribution between TB-positive and TB-negative patients. The IGRA test detected 23 positive cases and 178 negative cases, while the TST test revealed 77 positive cases and 124 negative cases. BCG vaccination status among the individuals was 99.0%.

Compared with QFT-Plus, TST showed high comparator-based sensitivity and a high NPV (97.6%) in this cohort. These measures indicate agreement with QFT-Plus results; they do not establish that TST correctly identifies or excludes LTBI because neither test is a definitive gold standard. The high proportion of BCG-vaccinated participants and the discordant results also mean that the observed TST-positive/QFT-Plus-negative results should not automatically be labeled false positives.

These findings are consistent with other research conducted in different parts of the world. In an analysis carried out in Germany, IGRA (QFT) was shown to be the superior test overall, particularly in cases of discordance between the two tests (TST vs. IGRA), and is likely the preferred option, especially in high-risk groups [11]. A study conducted among Polish healthcare workers also showed IGRA (QFT-GIT, QuantiFERON-TB Gold In-Tube) to be the superior test, as it is unaffected by BCG vaccination, which can generate false positives in TST [12]. The high BCG vaccination rate among our study population can explain the higher positive TST rate due to known cross-reactivity. A study comparing commercial IGRA assays and TST in the diagnosis of LTBI in Nepalese military recruits recently arrived in the UK also found that IGRAs were more sensitive, detecting LTBI in individuals who were TST-negative [13].

Furthermore, a cross-sectional study conducted at multiple non-tertiary care hospitals on healthcare workers who worked during the Hajj pilgrimage in Saudi Arabia found IGRA (QFT-GIT) to be the superior test, detecting more cases of LTBI where TST results were negative, particularly in healthcare workers with a history of close contact with TB patients. The poor agreement between the two tests (low kappa value) suggests that QFT-GIT may be more reliable in certain situations, particularly in higher-risk populations such as healthcare workers [14]. In addition, research comparing the two tests in healthcare workers in Riyadh found the QFT test to be superior, providing higher overall sensitivity and specificity than TST, particularly when the normal 10 mm cutoff was utilized. However, increasing the TST cutoff to 13 mm or greater enhanced specificity but decreased sensitivity [15].

A study conducted in India among 1,511 household contacts of pulmonary TB patients found that TST remains widely used in resource-limited settings; however, IGRA provided improved specificity in BCG-vaccinated populations [16].

According to various studies, IGRA offers several advantages over TST. A study conducted among 30-year-old healthcare workers revealed that the original QFT-G (QuantiFERON-TB Gold) and the updated QFT-GIT are more accurate and cost-effective than TST in identifying LTBI in healthcare workers, regardless of BCG vaccination status. IGRAs yield superior clinical outcomes and should be used to screen new healthcare workers. They also provide better specificity, reducing the risk of false positives, and require only a single blood draw, making them more convenient than TST, which requires two visits [17]. Furthermore, another review found that IGRAs are not affected by the booster effect that might occur with TST in individuals with prior TB exposure [18]. IGRAs provide quantitative results with more accurate information, whereas TST is based on visual evaluation of induration size [19].

Our results provide multifaceted implications for healthcare policy and clinical practice. The marked difference between IGRA (QFT-Plus) and TST positivity rates (11.4% vs. 38.3%) necessitates a re-evaluation of current tuberculosis screening protocols, particularly in regions with high BCG vaccination rates, as false positives may lead to unnecessary interventions and resource expenditure. While the high NPV (97.6%) of the TST test supports its use in initial screening, especially in resource-limited settings, the low PPV (26.0%) suggests the need for confirmatory IGRA testing for TST-positive cases. These findings contribute to global TB control efforts and research methodology in TB diagnostics, providing a framework for future comparative studies and highlighting the importance of considering local contexts, such as BCG vaccination rates, when developing and implementing TB screening strategies in healthcare settings. These findings carry direct implications for occupational tuberculosis screening protocols in healthcare settings with high BCG vaccination coverage. Given the moderate specificity and low PPV of TST observed in this cohort, a two-step screening algorithm - using TST as an initial, low-cost screen followed by confirmatory QFT-Plus testing for TST-positive healthcare workers - may reduce unnecessary treatment of false-positive results while preserving the operational simplicity of TST for large-scale periodic screening. The five-year follow-up finding of no progression to active tuberculosis among TST-positive/QFT-Plus-negative healthcare workers is reassuring but should be interpreted cautiously because the number of discordant participants was small and progression to active tuberculosis is an imperfect surrogate for LTBI status. The finding supports considering confirmatory IGRA testing before treatment decisions where clinically appropriate, rather than treating the discordant results as definitive evidence of either infection or no infection. Incorporating QFT-Plus confirmation into pre-employment and periodic occupational screening protocols could reduce the burden of unnecessary LTBI treatment, associated costs, and workforce disruption, while preserving sensitivity for detecting healthcare workers who require preventive therapy.

Limitations

This study has several limitations. The retrospective, single-center design and the absence of a definitive reference standard for LTBI are important. QFT-Plus was used as a comparator rather than a true gold standard; consequently, the reported sensitivity, specificity, predictive values, and ROC estimates are measures of concordance with QFT-Plus and may be affected by misclassification, spectrum effects, and imperfect agreement between the two tests. They should not be interpreted as the true diagnostic accuracy of TST for LTBI. The cohort was drawn predominantly from a single occupational health setting with a high proportion of BCG-vaccinated, non-Saudi healthcare workers, which may limit generalizability to other populations and settings. In addition, although a five-year follow-up was available to assess progression to active tuberculosis, the small number of discordant (TST-positive/QFT-Plus-negative) cases limits the statistical power of this analysis. The overall sample size was determined pragmatically based on feasibility rather than through a formal a priori power or precision calculation, which should be considered when interpreting the precision of the diagnostic accuracy estimates. Follow-up outcomes were ascertained from the hospital's electronic medical record rather than through active, standardized surveillance and could not capture care received outside the KFAFH system; data on preventive LTBI therapy uptake among discordant participants were also not systematically available, so the absence of progression to active tuberculosis in this subgroup cannot be fully attributed to a true absence of infection rather than to unrecorded treatment or unrecorded care obtained elsewhere. Although this study included a broad adult age range, the findings may not be generalizable to immunocompromised populations.

## Conclusions

The present study shows that TST and QFT-Plus yielded substantially different positivity rates in this highly BCG-vaccinated healthcare worker cohort. TST demonstrated higher comparator-based sensitivity and NPV but lower comparator-based specificity than QFT-Plus; these estimates reflect agreement with QFT-Plus and cannot be interpreted as definitive diagnostic accuracy because no gold standard for LTBI exists. QFT-Plus may nevertheless be operationally advantageous in BCG-vaccinated populations because it is less affected by BCG-related cross-reactivity, although the present study cannot establish that it is intrinsically more accurate for LTBI.

Additional validation of the Mantoux test is required to maximize its diagnostic accuracy in various populations. Given the high BCG vaccination rates in some settings and the limited specificity of the Mantoux test observed here, QFT-Plus confirmatory testing may have a useful role in occupational LTBI screening algorithms, particularly for TST-positive healthcare workers in highly BCG-vaccinated populations; the fourth-generation QFT-Plus assay also offers logistical advantages, including a single blood draw and quantitative, less subjective results. However, because this study cannot establish the absolute diagnostic accuracy of either test and was not designed to evaluate treatment decisions, any change to screening or treatment protocols should be considered in the context of current national and international TB guidelines rather than on the basis of these findings alone. Decisions to withhold preventive therapy from TST-positive/QFT-Plus-negative individuals should not rest solely on the absence of progression to active tuberculosis observed in this small discordant subgroup. A five-year follow-up demonstrated no progression to active tuberculosis among individuals with discordant TST-positive/IGRA-negative results.

## References

[1] Houben RM, Dodd PJ (2016). The global burden of latent tuberculosis infection: a re-estimation using mathematical modelling. PLoS Med.

[2] Hsu D, Irfan M, Jabeen K (2020). Post tuberculosis treatment infectious complications. Int J Infect Dis.

[3] Seung KJ, Keshavjee S, Rich ML (2015). Multidrug-resistant tuberculosis and extensively drug-resistant tuberculosis. Cold Spring Harb Perspect Med.

[4] (2026). Ministry of Health, Saudi Arabia. National tuberculosis program manual. https://www.moh.gov.sa/documents/national-tb-program-3.21.pdf.

[5] Al-Sahafi A, Al-Sayali MM, Mandoura N (2021). Treatment outcomes among tuberculosis patients in Jeddah, Saudi Arabia: results of a community mobile outreach directly observed treatment, short-course (DOTS) project, compared to a standard facility-based DOTS: a randomized controlled trial. J Clin Tuberc Other Mycobact Dis.

[6] Al-Hajjaj MS (2000). The outcome of tuberculosis treatment after implementation of the national tuberculosis control program in Saudi Arabia. Ann Saudi Med.

[7] Alsdurf H, Empringham B, Miller C, Zwerling A (2021). Tuberculosis screening costs and cost-effectiveness in high-risk groups: a systematic review. BMC Infect Dis.

[8] Sousa S, Rocha D, Silva JC (2021). Comparing the cost-effectiveness of two screening strategies for latent tuberculosis infection in Portugal. Pulmonology.

[9] (2026). World Health Organization. Global tuberculosis report. https://www.who.int/teams/global-tuberculosis-programme/tb-reports/global-tuberculosis-report-2024.

[10] Auguste P, Tsertsvadze A, Pink J (2017). Comparing interferon-gamma release assays with tuberculin skin test for identifying latent tuberculosis infection that progresses to active tuberculosis: systematic review and meta-analysis. BMC Infect Dis.

[11] Nienhaus A, Schablon A, Diel R (2008). Interferon-gamma release assay for the diagnosis of latent TB infection - analysis of discordant results, when compared to the tuberculin skin test. PLoS One.

[12] Szturmowicz M, Broniarek-Samson B, Demkow U (2021). Prevalence and risk factors for latent tuberculosis in Polish healthcare workers: the comparison of tuberculin skin test and interferon-gamma release assay (IGRA) performance. J Occup Med Toxicol.

[13] O'Shea MK, Fletcher TE, Beeching NJ (2014). Tuberculin skin testing and treatment modulates interferon-gamma release assay results for latent tuberculosis in migrants. PLoS One.

[14] Bukhary ZA, Amer SM, Emara MM, Abdalla ME, Ali SA (2018). Screening of latent tuberculosis infection among health care workers working in Hajj pilgrimage area in Saudi Arabia, using interferon gamma release assay and tuberculin skin test. Ann Saudi Med.

[15] AlHamdan NAE, Abbas MAF, AlQahtani MS, Klantan S, Fiala LA, Elghazali G (2013). Comparison of tuberculin skin test (TST) and QuantiFERON test (QFT) for detection of latent TB infection among health care workers (HCWs) in a tertiary care hospital in Riyadh, Saudi Arabia. Life Sci J.

[16] Sharma SK, Vashishtha R, Chauhan LS, Sreenivas V, Seth D (2017). Comparison of TST and IGRA in diagnosis of latent tuberculosis infection in a high TB-burden setting. PLoS One.

[17] de Perio MA, Tsevat J, Roselle GA, Kralovic SM, Eckman MH (2009). Cost-effectiveness of interferon gamma release assays vs tuberculin skin tests in health care workers. Arch Intern Med.

[18] Starke JR (2020). Tuberculin skin test versus the interferon-γ release assays: out with the old, in with the new. Pediatrics.

[19] Regatieri A, Abdelwahed Y, Perez MT, Bush LM (2011). Testing for tuberculosis: the roles of tuberculin skin tests and interferon gamma release assays. Lab Med.

